# Cancer Prevention by Natural Products Introduced into the Diet—Selected Cyclitols

**DOI:** 10.3390/ijms21238988

**Published:** 2020-11-26

**Authors:** Karol Wiśniewski, Marcin Jozwik, Joanna Wojtkiewicz

**Affiliations:** 1 Department Pathophysiology, School of Medicine, University of Warmia and Mazury, 10-082 Olsztyn, Poland; wisniewski.karol@op.pl; 2 Department of Gynecology and Obstetrics, School of Medicine, Collegium Medicum University of Warmia and Mazury, 10-561 Olsztyn, Poland; marcin.jozwik@uwm.edu.pl

**Keywords:** cancer, cyclitols, IP6, inositol, chemoprevention, plant foods

## Abstract

Cancer is now the second leading cause of death worldwide. It is estimated that every year, approximately 9.6 million people die of oncologic diseases. The most common origins of malignancy are the lungs, breasts, and colorectum. Even though in recent years, many new drugs and therapeutic options have been introduced, there are still no safe, effective chemopreventive agents. Cyclitols seem poised to improve this situation. There is a body of evidence that suggests that their supplementation can decrease the incidence of colorectal cancer, lower the risk of metastasis occurrence, lower the proliferation index, induce apoptosis in malignant cells, enhance natural killer (NK) cell activity, protect cells from free radical damage, and induce positive molecular changes, as well as reduce the side effects of anticancer treatments such as chemotherapy or surgery. Cyclitol supplementation appears to be both safe and well-tolerated. This review focuses on presenting, in a comprehensive way, the currently available knowledge regarding the use of cyclitols in the treatment of different malignancies, particularly in lung, breast, colorectal, and prostate cancers.

## 1. Cyclitols

Throughout the ages, medicine has relied on natural products, most of which come from plants. However, ancient scientists did not have knowledge of which chemical particles found in plants were responsible for the positive effects of treatment. Currently, thanks to advanced technologies, we are able to extract or synthesize the chosen substances. Cyclitols are natural, widespread, and very promising. This group of compounds, from a chemical point of view, is based on a cycloalkane chain that contains at least three hydroxyl groups, each attached to a different ring carbon. What is interesting about cyclitols is that they are involved in numerous biological processes at a molecular level, so their supplementation can provide a wide range of effects. To date, their role in membrane biogenesis, signal transduction, channel physiology, osmoregulation, phosphate storage, cell wall formation, and antioxidant activity has been confirmed. The most common cyclitols in eukaryotic cells are inositols (Ins) [1,2].

**Figure 1:**
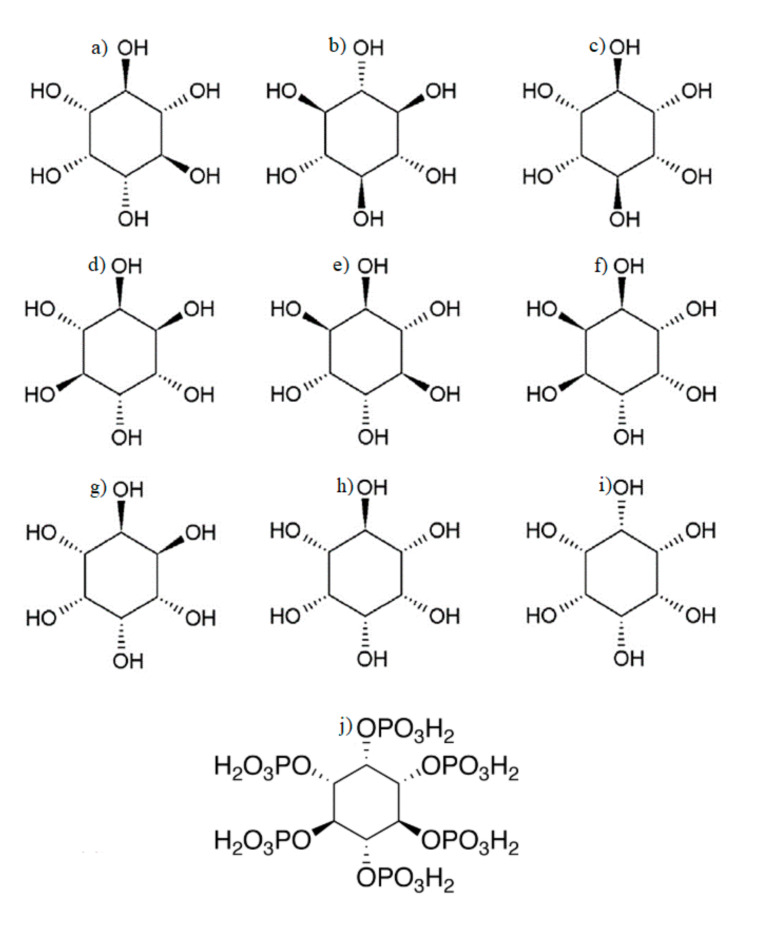
Structural formulas of (**a**) myo-inositol (MI); (**b**) scyllo-inositols (Ins); (**c**) mucco-Ins; (**d**) D-chiro-Ins; (**e**) L-chiro-Ins; (**f**) neo-Ins; (**g**) allo-Ins; (**h**) epi-Ins; (**i**) cis-Ins; and (**j**) phytic acid (IP6).

Inositols (Figure 1) are a family of compounds derived from C6 sugar alcohol, with the empirical formula C_6_H_12_O_6_ (1.2,3.4,5.6-cyclohexanol). There are nine known forms of inositol: myo-inositol (MI), scyllo-inositol, muco-inositol, epi-inositol, allo-inositol, cis-inositol, neo-inositol, l-chiro-inositol, and d-chiro-inositol [3]. MI is the dominant form of Ins in intracellular content [4].

MI is, therefore, a widespread molecule, whose supplementation appears to have several positive effects. The most common and easiest method of supplementation of MI and other cyclitols appears to be oral. MI can be consumed by humans in three different forms: free form, inositol-containing phospholipids, and, most frequently, as a phytic acid (IP6) [5]. The largest amount of IP6 can be found in almonds (9.4% of dry weight), walnuts (6.7% of dry weight), and Brazil nuts (6.3% of dry weight) [6]. Among fruits, cantaloupe and citruses (with the exception of lemons) have a high concentration of MI [7]. A 120 g portion of grapefruit juice can provide about 470 mg of MI [8]. However, a typical 2500 kcal American diet provides only about 900 mg of MI per day, whereas a well-balanced diet can provide up to 1500 mg of MI per 1800 kcal [8].

Almost all of the free MI ingested (99.8%) is absorbed in the human gastrointestinal tract. This uptake is possible due to active transport, which occurs against the concentration gradient in a Na+-dependent manner [5,9]. Importantly, glucose or other sugars can significantly reduce MI uptake in a noncompetitive manner [3]. When it comes to the plasma levels of MI achieved by oral intake, it is currently known that supplementation with 4 g of MI powder results in a maximum plasma concentration (Cmax) of 45 µmol/L, whereas supplementation with 2 g MI powder results in a Cmax of 36.3 µmol/L [10]. In normal and healthy subjects, the plasma levels of MI were found to be approximately 30 mM, with a 22 min half-life [7]. MI is also present in serum as phytic acid, with a plasma level of approximately 0.1–0.4 mM. MI can be found in phospholipids or circulating lipoproteins [7].

MI is also synthesized endogenously—primarily in the kidneys, which produce about 4 g of MI per day [3]. Comparing the amounts of MI provided by the diet to the amounts synthesized endogenously, it may seem that supplementation of MI is futile. However, it was proven that rats fed with an MI-free diet had significantly lower concentrations of MI in every tissue, except for the brain [11]. This study found grounds for exogenous supplementation of MI. The kidneys appear to be the most important organ in MI catabolism to d-glucuronic acid through myo-inositol oxygenase [7].

Although MI is involved in numerous documented and possibly unknown processes, supplementation with MI appears to be safe and well-tolerated. The lethal dose that caused the death of 50% of a group of test animals (LD50) of orally administrated MI in mice was about 10,000 mg/kg body weight [10]. In humans, orally administrated doses are typically up to 18 g per day. The side effects of MI supplementation are rare and mild and mainly encompass gastrointestinal symptoms [12]. The safety of MI has been confirmed by its use in infant milk powders, with a generally recognized as safe (GRAS) status [13].

## 2. Cancers

Neoplastic tumors involve a wide group of disorders originating from different tissues. They are characterized by the uncontrollable growth of abnormal, immature cells capable of invading adjoining tissues and/or spreading to other organs of the body.

Malignancies are the second leading cause of death worldwide, with an estimated 9.6 million oncologic fatalities in 2018. The most common origins of cancer in the male population were the lungs, prostate, colorectum, stomach, and liver. In the female population, the prevailing types were breast, colorectal, lung, cervical, and thyroid cancer [14].

It is currently understood that many cancers are influenced by environmental factors such as smoking and alcohol intake, as well as inappropriate diet [14]. Eliminating these and other risk factors is one of the best ways to reduce the number of deaths caused by cancers. Although prophylactic methods, like vaccines against HPV to prevent cervical cancer [14,15], have been tested, to date, there is no safe chemopreventive agent recommended worldwide for the treatment of malignancies.

The treatment of cancer is a sophisticated, multidisciplinary, and costly field of medicine. The introduction of new drugs has increased the life span of people affected by different tumors; however, the overall cure rate remains unsatisfactory, and there is still a need for new treatment options and new medicines. An important problem is the issues caused by the toxicity of oncological drugs, in particular, neutropenia [16] and thrombocytopenia [17]. Even though new therapeutic options have been introduced to fight against the side effects of cancer treatment, the adverse effects are a critical problem in oncology. Cyclitols may play an important role, as a supplement to regular treatment, by improving the cure rate and decreasing adverse events.

## 3. Lung Cancer and Cyclitols

Lung cancer is the most common malignancy globally and is the leading cause of cancer-related deaths. According to WHO data, there were over 2 million new cases of lung cancer and over 1.76 million fatalities in 2018 [18]. Considering the high mortality, there have been numerous studies carried out in the search for an effective agent against lung cancer [19]. Attempts concentrating on the use of conventional X-ray scanning or cytological sputum tests did not help to reduce the morbidity of lung cancer [20]. Currently, the only effective method of decreasing the mortality of lung cancer is low-dose computed tomography (CT). The National Lung Screening Trial (NLST) showed that low-dose chest CT when compared to X-ray examination, resulted in a 20% reduction in lung cancer mortality among high-risk individuals (aged 55–74 years and with more than 30 pack-years of smoking history) [21]. Despite limited diagnostic measures and unsatisfactory treatment results, to date, no nutrient has been implemented as a lung tumor prevention agent.

MI may be a possible chemopreventive agent. The data collected by Enstensen et al. showed that mice fed with a 3% MI diet had 40% fewer lung adenomas than the control. Research has demonstrated that a diet with both 3% MI and 0.5 µg/g dexamethasone had an additive effect on the inhibition of pulmonary adenoma formation [22]. These positive effects on reducing tumor multiplicity by MI supplementation are supported by other studies [23,24,25]. A combination of MI and *N*-acetyl-S-(*N*-2-phenethylthiocarbamoyl)-l-cysteine (PEITC-NAC) may be more effective at reducing lung tumor multiplicity compared with supplementation of any of these substances alone [24]. Myo-inositol supplementation appears to reduce the activity of IL-6 related pathways, including STAT3 phosphorylation [25]. IL-6 related pathway activation is currently believed to be involved in tumor progression [26].

The results of animal studies led to studies in humans. A phase I study for lung cancer chemoprevention showed that MI supplementation should be investigated as a chemopreventive agent against lung cancer. A significantly increased rate of regression of pre-existing dysplastic lesions was observed in the MI supplementation group [12]. Researchers also found that, after MI treatment, there was a significant reduction in Akt and ERK phosphorylation in dysplastic lesions, but not in hyperplastic or metaplastic ones [27]. Unfortunately, a randomized phase IIb trial of MI used as a chemopreventive agent in smokers did not prove the effectiveness of MI. There was no statistically significant regression or progression of bronchial lesions for the MI group versus the placebo. However, a significant reduction of the IL-6 level in BAL and a significant decrease in the gene expression signature reflective of PI3K activation within the cytologically normal bronchial airway epithelium was seen among complete responders in the MI group. Such a decrease of PI3K activation was not observed among the patients with a complete response in the placebo group [28].

In summary (Table 1), despite the positive effects of MI administration in lung cancer models in animal trials and the evidence that MI reduces the levels of IL-6 and may decrease the activation of PI3K signaling pathways, the results obtained from trials conducted on humans do not support MI usage alone as a possible chemopreventive agent against lung cancer. Based on the results of animal studies, the combination of MI and PEITC-NAC or MI and dexamethasone may have a greater impact on lung cancer chemoprevention. Further studies to evaluate the possible effects of a combination of MI with other substances in lung cancer prevention are needed.

## 4. Breast Cancer and Cyclitols

Breast cancer is the most common malignancy in the female population globally, and the second most common malignancy in both sexes [14]. There were over 2 million new cases of breast cancer and over 626,000 fatalities (fifth most-common cause of cancer mortality globally in both sexes) in 2018 [14]. This remarkable difference between the number of new cases of breast cancer and mortality was attributed to the introduction of screening programs (mammography) and the development of new chemotherapy regimens, which have changed the treatment of breast cancer in recent years. Now, new substances to prevent breast cancer, support its treatment and reduce side effects are needed.

Animal trials have provided evidence that IP6 and inositol may be promising chemopreventive agents. The data collected by Shivapurkar et al. showed that the administration of 2% IP6 with a low-fiber diet resulted in a reduction of breast tumor incidence in rats [29]. Similar results of IP6 efficiency in preventing breast cancer were reported in other studies [30,31]. There is also evidence that MI, either alone or in combination with IP6, may have chemopreventive properties against breast cancer [30].

The results of in vitro trials showed that treatment with IP6 was able to reduce the growth of cancer cells, suppress DNA synthesis, increase the expression of lactalbumin, which is associated with luminal cell differentiation [32], arrest cancer cells in the G0/G1 phase [33], decrease the S phase and level of KI-67 expression in cancer cell lines [33], reduce adhesion and motility [34,35], increase the expression of antiproliferative agent PKCδ, increase the activity of p27Kip1, decrease Erk1/2 and Akt activity, and reduce pRb phosphorylation [36]. IP6 may have a synergistic effect on breast cancer treatment with adriamycin and tamoxifen [37]. In vitro trials also showed that the exposure of breast cancer cell lines to inositol resulted in reduced PI3K and phosphorylated Akt activity, decreased SNAI1 expression, increased levels of E-cadherin and β-catenin, and reduced motility, invasiveness, and cytoskeleton stabilization [38].

Human trials demonstrated a potentially positive role of IP6 and MI in the treatment of breast cancer. Bačić et al. reported that supplementation of IP6 + inositol in the treatment of invasive ductal breast cancer with the FEC chemotherapy protocol resulted in fewer side effects and better quality of life as well as a better functional status of supplemented patients as compared to controls. A remarkable observation was that IP6 + inositol-treated patients had smaller decreases in their white blood cell count and platelet count after chemotherapy, while a more significant decrease was noted in the control group [39]. This is an important finding because neutropenia is a common reason to reduce or withhold chemotherapy [40]. Similar results were obtained by Proietti et al. [41]. 

The group found that the topical usage of 5 g of 4% IP6 as a sodium salt (200 mg of IP6), applied once a day on the breast after lumpectomy during CMF chemotherapy, resulted in fewer side effects, fewer postponed chemotherapy cycles and an improvement in the quality of life and functional status [41].

There is evidence that MI may be a possible therapeutic option in the treatment of other breast conditions, like high breast density [42], which is recognized as a risk factor of breast cancer [43]. MI appears to have a positive effect on the management of breast fibroadenomas [44], a common finding among young women, with a very low risk of cancer transformation [45]. To date, no preventive treatment has been implemented for asymptomatic fibroadenomas, although some grow and require intervention due to clinical symptoms or a bulky size [46]. In such cases, a possible conservative treatment option may be an anti-estrogen drug instead of surgical management [46]. Pasta et al. found supplementation with 400 mg MI (+Boswellia 100 mg and Betaine 350 mg) twice a day for six months resulted in a reduction of the fibroadenoma median volume. There were no side effects recorded during the study [44].

In brief, supplementation with IP6 alone and with IP6 + inositol both seem to exert positive effects in the management of breast cancer, particularly decreasing the side effects of chemotherapy such as neutropenia. Protective molecular changes in gene expression were noted in trials on animals. There is evidence that MI may be a possible therapeutic option in the management of increased breast density or fibroadenomas. The effects of inositol on managing breast conditions are summarized in Table 2.

## 5. Colorectal Cancer and Cyclitols

Colorectal cancer is the third most common malignancy globally. In 2018, there were almost 1.85 million new cases of colorectal cancer, with over 880,000 fatalities, making it the second leading cause of death among cancers. In terms of chemoprevention, aspirin treatment decreases the risk of colorectal cancer, although the side effects of its long-term use make it unsuitable for large-scale prophylactic programs [47].

IP6 and inositol appear to exhibit chemopreventive properties against colorectal cancer. The data collected by Shamsuddin et al. showed that supplementation with 1% of Na-IP6 in drinking water resulted in a significant reduction of tumor incidence and a decrease in the mitotic rate in the colonic crypts [48]. These results are supported by other studies [30,50,51,52,53,54]. Supplementation with both IP6 and green tea had a synergistic effect on reducing the multiplicity of tumors (particularly 2% IP6 and 2% green tea) [49]. There are also reports indicating that IP6 and Ins have an immunostimulating effect on NK-cells [50,51]. This is an important finding because it is currently believed that NK-cell activity is in reverse correlation with tumor incidence and that supplementation with both IP6 and Ins may have a synergistic effect on increasing NK activity [50].

Treatment with IP6 also appears to result in inhibitory changes in cancer cell lines, such as a significant restriction of growth, DNA synthesis, and proliferation [52], a decrease of the KI-67 index [53], and an increase of the apoptotic index [54]. The molecular changes after IP6 supplementation involve a reduction of the expression of PI3K, Akt and pAk, collagen IV, fibronectin, laminin, integrin β1, MMP-9, VEGF, bFGF, TGF-β [53], p21Waf1/Cip1 [54], and an increase in caspase-9 activity [53]. Again, treatment with both Ins and IP6 appears to have a synergistic effect [53]. Complementary results were obtained by Schröterová et al., who showed that exposure to IP6 significantly decreased the levels of MMP-2, MMP-9, I-Cam1, EpCam, and N-cadherin. Cell migration was significantly reduced after IP6 treatment [55].

In summary, IP6 demonstrated chemopreventive properties against colorectal cancer. Research showed that both IP6 and Ins enhanced NK-cell activity, which was inversely associated with tumor occurrence. There is evidence that IP6 and inositol supplementation may reduce the expression of proteins, enzymes, and factors that are crucial for the occurrence of metastasis, which leads to the conclusion that IP6 and Ins may reduce the number of colorectal cancer metastases. A summary of the effects of cyclitols on managing colorectal cancers is presented in Table 3.

## 6. Prostate Cancer and Cyclitols

The prevalence of prostate cancer has increased in recent years. It is now the most common malignancy in elderly men in the western world [59]. The risk of developing prostate cancer is mainly associated with age—the probability increases from 0.005% in men younger than 30 to around 50% in men older than 70 years [60,61]. The lifetime risk of developing prostate cancer is currently estimated at 16.7% (one in six men) [62]. Although only a small number of patients will experience fully invasive prostate cancer, the high prevalence of this malignancy shows the need for new drugs, therapeutic options, and efficient chemopreventive agents.

Cyclitols, in particular IP6, are a good candidate for possible chemopreventive and therapeutic options for prostate cancer (Table 4). A study conducted by Shamsuddin et al. on PC-3 human prostate cancer cells showed that treatment with IP6 might inhibit both growth and DNA synthesis. IP6 treatment increased the expression of HLA-1 class antigens and increased prostatic acid phosphatase (PAP) activity, indicating cell differentiation [63]. Research has reported that IP6 can induce G0/G1 cell cycle arrest of treated cells [64]. A study conducted by Singh et al. showed antiproliferative changes in the protein expression and activity in prostate cancer cells after treatment with IP6. In one report, mice that were injected with DU145 cells and then treated with 2% IP6 in drinking water had significantly reduced tumor weights. IP6 significantly decreased the proliferation index and increased the apoptotic rate. The microvessel density in prostate tumor xenografts was significantly reduced after IP6 treatment [65].

Another cyclitol that appears to have a positive effect on prostate cancer treatment is d-pinitol. A study by Lin et al. showed that there was a significant decrease in cell migration and invasion after treatment with d-pinitol. These observations were supported by significantly lower integrin αvβ3 expression in the experimental group. Significant decreases in the levels of p-FAK and p-p65 and in the activity of c-Src kinase and NF-κβ luciferase after d-pinitol treatment were noted. However, in contrast to IP6, d-pinitol does not appear to increase the apoptotic rate of prostatic cancer cells [63].

In summary, IP6 demonstrates the properties of a possible chemopreventive agent against prostate cancer and may be implemented in the future. In contrast, d-pinitol appears to be a cyclitol that may find use in more advanced stages of the disease. The small number of studies conducted indicates the need for further research to evaluate the value of these cyclitols in prostate cancer treatment.

## 7. Cyclitols and Other Cancers

There are reports showing the positive effects of cyclitols on other types of cancers (Table 4). A study conducted by Somasundar et al. showed that treatment of pancreatic cancer cells (MIAPACA and PANC1) with IP6 resulted in significant growth inhibition and an increased apoptotic rate [66]. These results were supported by a study by McMillan et al., which showed that the IP6 effect on growth inhibition might be increased by catechin. The latter trial showed that treatment with IP6 reduced the level of VEGF, and synergism with catechin was noted. However, in this study, no increased apoptosis was noted in the IP6-treated group, but a synergism was noted between catechin and IP6 in inducing apoptosis [67]. Clinical trials are being performed to evaluate the possible benefits of the treatment of hepato-pancreato-biliary neoplasm with myo-inositoltrispyrophosphate (ITPP) [72].

Liver cancer is another type of malignancy that cyclitols appear to have positive effects on. A study conducted by Lee et al. showed that supplementation with either IP6 or In resulted in a significantly lower number of preneoplastic lesions. A synergistic effect was noted between IP6 and In. The next finding of the study was that in IP6- and/or In-treated groups, there was a significantly lower level of lipid peroxidation, a known factor enhancing cancer development [68]. A report by Nishino et al. demonstrated that supplementation with both MI and β-cryptoxanthin in mandarin orange juice and carotenoid capsules was able to reduce hepatocellular cancer incidence by 81% in patients with chronic viral hepatitis and cirrhosis. In this same experiment, supplementation with only carotenoid capsules reduced cancer incidence by only about 50% [69].

A study conducted by Ren et al. showed that treatment with IP6 might have positive effects on osteosarcoma. In this trial, it was not only shown that IP6 can inhibit the proliferation rate and cell growth in the K7M2 and MG63.3 cell lines, but also that IP6 treatment was able to increase the survival rate in a mouse model of osteosarcoma metastases [70].

Finally, a case report indicated that treatment with both IP6 and Ins resulted in the complete clinical and radiological remission of melanoma [71].

## 8. Cyclitols and Other Disorders

The supplementation of cyclitols appears to have positive effects on many other disorders. The effects on polycystic ovary syndrome (PCOS) involve a decrease in the BMI [73], a return of spontaneous ovary function, improved fertility [73], positive hormonal changes [74,75], and a decrease in the HOMA-IR index, as well as positive lipid profile changes [76]. Cyclitols appear to be useful in the treatment of metabolic syndrome and diabetes [2]. There is also a report suggesting that MI treatment may be a game-changing option for people diagnosed with Hashimoto’s thyroiditis [77].

## 9. Conclusions

Cancer is one of the most serious problems in medicine. Although many new drugs and therapies have been introduced in recent years, malignancies are still the second leading cause of death worldwide. One of the factors that may be responsible for such unsatisfactory statistics is that there is no known safe chemopreventive agent that may be used on a large scale for most cancers. Cyclitols may bridge the gap in this area. There is evidence that cyclitols may, in the future, become one of the elements of cancer treatment—both as an antitumor agent and as an agent helping to reduce the side effects of other therapies. The antineoplastic properties of cyclitols appear to be mainly due to the reduction of the activity of the IL-6- and STAT3-related pathways, stimulating NK cell activity, positive molecular changes in gene expression, and cytoskeleton stabilization. To fully recognize the potential of different cyclitols in both chemoprevention and the treatment of cancers, further and larger-scale human trials are required.

## Figures and Tables

**Table 1:** Antineoplastic activity of cyclitols in lung/bronchus cancer models.

Study	Cyclitol	Human/Animal Species	Results
Enstensen et al. (1993) [22]	MI	A/J mouse	3% MI diet, a 40% reduction of the number of lung adenomas multiplicity 3% MI diet + 0.5 µg/g dexamethasone, an additive effect on the inhibition of pulmonary adenomas formation
Hecht et al. (2001) [23]	MI	A/J mouse	reduction of tumor multiplicity (reduction of 28.9% and 33.0% in doses 1% and 0.5% (experiment 1), reduction of 48.4% in dose 1% (experiment 2) significant MI dose trend for inhibition of tumor multiplicity (*p* < 0.0001)
Hecht et al. (2002) [24]	MI	A/J mouse	the MI diet reduced lung tumor multiplicity MI + PEITC-NAC combination was more effective than the use of MI alone (*p* = 0.0015) or PEITC-NAC (*p* < 0.0001); MI was more effective than PETC-NAC (STAT3 < 0.0001) Increasing duration of treatment was significantly correlated with decreasing tumor multiplicity (for MI + PEITC-NAC– *p* = 0.0045; for MI– *p* = 0.015)
Lam et al. [12] (2006)	MI	Human	a significant increase in the rate of regression of pre-existing dysplastic lesions (91% vs. 48%, *p* = 0.014)
Han et al. [27] (2009)	MI	Human	MI significantly decreased the Akt and ERK phosphorylation in dysplastic lesions (*p* < 0.01 and *p* < 0.05, respectively) in vitro, MI decreased the endogenous and tobacco carcinogen-induced activation of Akt and ERK in immortalized, human bronchial epithelial cells
Lam et al. [28] (2016)	MI	Human	a significant reduction of IL-6 in BAL (*p* = 0.03) a decrease in a gene expression of PI3K activation within the cytologically normal bronchial airway epithelium (*p* = 0.002) no statistically significant difference between MI and placebo arms in response or progression of bronchial lesions
Unver et al. (2018) [25]	MI	Mouse model (Ccsp^Cre/+^; Kras ^LSL-G12D/+^)	reduction in the number of lung tumors (46 ± 2.7 in the control group vs. 22.0 ± 2.0 in the MI group) *p* < 0.05 statistically significantly reduced levels of IL-6 and LIF reduced number of macrophages in the tumor (*p* = 0.004)

**Table 2:** Antineoplastic activity of cyclitols in breast tumors.

Study	Clinical status	Cyclitol	Results
Shivapurkar et al. (1995) [29]	MUN-induced rat mammary cancer	IP6	Significant reduction of mammary tumor incidence at both 11 and 32 weeks of the experiment
Vucenik et al. (1995) [30]	DMBA-induced rat mammary cancer	IP6; Ins; IP6 + Ins	Significant reduction of tumor incidence in a group treated with: IP6 (*p* = 0.02), Ins (*p* < 0.05), or IP6 and Ins (*p* < 0.05) compared to controls 58% reduction of tumor multiplicity in IP6 + Ins group compared to controls (1.8 ± 0.1 versus 3.1 ± 0.3, respectively) (*p* < 0.05)
Shamsuddin et al. (1996) [32]	Human breast cancer cell lines MDA-MB-231 and MCF-7	IP6	Dose-dependent growth inhibition of both cell lines, suppression of DNA synthesis Increased expression of lactalbumin (associated with luminal cell differentiation) up to 22-fold compared to controls
El-Sherbiny et al. (2001) [33]	Human breast cancer cell lines MDA-MB-231 and MCF-7	IP6	Treatment with IP6 decrease S phase and arrest cells in the G0/G1 phase Significant decrease in the percentage of Ki-67 expression in IP6-treated cells (*p* < 0.01)
Tantivejkul et al. (2003) [34]	Human breast cancer cell line: MDA-MB-231	IP6	65% reduction of cell adhesion of IP6-treated cells to fibronectin (*p* = 0.002), and 37% reduction of cell adhesion to collagen (*p* = 0.005) Reduced number of migrating cells and the distance of cell migration (*p* < 0.001) Absence of lamellipodia structure in IP6-treated cells (*p* = 0.001) IP6 treatment inhibited MMP-9 secretion (*p* = 0.006)
Tantivejkul et al. (2003) [35]	Human breast cancer cell line: MDA-MB-231	IP6	Decreased expression of integrin heterodimers: alpha2beta1 (collagen receptor), alpha5beta1 (fibronectin receptor) and alpha5beta3 (vitronectin receptor) (*p* < 0.005)
Tantivejkul et al. (2003) [37]	MCF-7, MDA-MB 231 and adriamycin-resistant MCF-7 (MCF-7/Adr) human breast cell lines	IP6	MCF-7/Adr was the most sensitive cell line for IP6 treatment (IC50 of 1.26 mM), while MCF-7 cell line was the least sensitive one (IC50 = 4.18 mM) Synergism effect of growth suppression when IP6 was administered prior to adriamycin (especially against MCF-7 cells (*p* < 0.0001) Synergism with tamoxifen in all three lines
Vucenik et al. (2005) [36]	MCF-7 human breast cancer cell line	IP6	3.1-fold increased expression of antiproliferative PKCδ (*p* = 0.0002) Decrease in Erk1/2 and Akt activity (*p* < 0.05) Increased p27^Kip1^ and marked reduction of pRb phosphorylation (*p* < 0.05)
Bačić et al. (2010) [39]	Ductal invasive breast cancer during FEC chemotherapy protocol	IP6	Patients treated with IP6 and Inositol had a significantly higher quality of life than patients from the placebo group, significantly higher functional score compared to the placebo group (87.9 versus 56.3; *p* = 0.0003), and lesser side symptoms (13.5 versus 33.8 on the symptomatic scale; *p* = 0.04) No leukocyte or platelet drop in the IP6- and inositol-treated group (the drop in leukocytes and platelets was significant in the control group)
Pasta et al. (2015) [42]	Mammographic breast density (premenopausal women)	MI	Significant reduction of breast density in the MI group compared to the placebo group (60% versus 9%), *p* < 0.001 Significant pain reduction after treatment in 13 out of 15 (86.7%) women with high breast density
Pasta et al. (2016) [44]	Breast fibroadenomas	MI	Reduction of fibroadenoma median volume for 17.86% (*p* = 0.005) versus 5.96% in the placebo group (*p* = n.s.) 38.88% of patients from the MI arm had a reduction of fibroadenoma volume, compared to 17.85% in the placebo arm No patients from the placebo or experimental arm showed signs of worsening
Dinicola et al. (2016) [38]	Human breast cancer cell lines MDA-MB-231 and ZR-75	Inositol	Reduction of PI3K activity by 40% (*p* < 0.001) and phosphorylated Akt by 35% (*p* < 0.05) after Ins treatment, compared to controls Ins treatment increased by 7-fold the level of E-cadherin (*p* < 0.001) and doubled the level of β-catenin (*p* < 0.01), compared to controls Decreased SNAI1 expression after Ins treatment (*p* < 0.001) Ins treatment reduced both motility (*p* < 0.001) and invasiveness (*p* < 0.05) of breast cancer cells No detectable formation of lamellipodia and filopodia was observed in Ins-treated cells Cytoskeleton stabilization in Ins-treated cells.
Proietti et al. (2017) [41]	Ductal breast cancer stages II‒III postoperative (lumpectomy); during polychemotherapy CMF	IP6	Significant reduction of chemotherapy side effects in IP6-treated group compared to controls (12 ± 10 versus 45.81 ± 10.0; *p* ≤ 0.001) No leukocytes or platelets drop in the IP6-treated group (reduction of leukocytes and platelets was significant in the control group) One-third the number of postponed chemotherapy cycles in the IP6-treated group compared to the control group Significant improvement of quality of life in the IP6-treated group Significant improvement of functional status in the IP6-treated group

**Table 3:** Antineoplastic activity of cyclitols in colon cancer.

Study	Clinical Status	Human/Animal Species	Cyclitol	Results
Shamsuddin et al. (1988) [48]	AOM-induced colon cancer	Rat	IP6	Significant reduction of tumor incidence in the IP6-treated group Significantly lower mitotic rate in the IP6-treated group
Shamsuddin et al. (1989) [56]	AOM-induced colon cancer	Rat	IP6	Even though the treatment with IP6 was introduced five months after carcinogen administration, there was a significant reduction in the number of tumors and tumor volume, and a lower mitotic rate was observed
Baten et al. (1989) [50]	DMH-induced colon cancer	Mouse	IP6	Significant increase in NK activity after IP6, Ins, and Ins + IP6 treatment Increased NK activity was correlated with reduced tumor incidence
Ullah et al. (1990) [57]	AOM-induced colon cancer	Rat	IP6	Supplementation with 1% IP6 in drinking water reduced tumor prevalence by 52.2%, frequency by 55.8%, and size by 62.3% 0.1% IP6 significantly reduced tumor size (by 71%)
Pretlow et al. (1992) [49]	AOM-induced colon cancer	Rat	IP6	Decreased incidence of tumors—25% in a group treated with IP6 versus 83% in a control group (*p* = 0.0045)
Yang et al. (1995) [52]	HT-29 human colon carcinoma cells		IP6	Statistically significant growth inhibition at 1 mM IP6 concentration and significant inhibition of DNA synthesis Decreased level of proliferation marker PCNA after 48 h
Shivapurkar et al. (1995) [29]	AOM-induced colon cancer	Rat	IP6	Significant reduction of colon tumor incidence at both 11 and 32 weeks of the experiment.
Challa et al. (1997) [58]	AOM-induced colon cancer	Rat	IP6	IP6 significantly reduced the incidence of aberrant crypt foci A synergy effect between IP6 and green tea was observed in reducing tumor multiplicity (especially with 2% of IP6 and 2% of green tea)
El-Sherbiny et al. (2001) [33]	HT-29 human colon carcinoma cells		IP6	Treatment with IP6 decreased S phase and arrest cells in the G0/G1 phase Significant decrease in the percentage of Ki-67 expression in IP6-treated cells (*p* < 0.01)
Zhang et al. (2005) [51]	DMH-induced colon cancer	Rat	IP6	Significant increase in blood NK activity in the IP6-treated group Significant reduction in the multiplicity and size of tumors in the IP6-treated group Significantly higher survival rate in the IP6-treated group
Liu et al. (2015) [53]	HT-29 human colon carcinoma cells		IP6	Significant inhibition of proliferation after exposure to IP6 (for 12 and 24 h) Significant reduction of expression of PI3K, Akt, and pAkt in IP6-treated cells Significant increase in the expression of caspase-9 in IP6-treated cells
Kapral et al. (2017) [54]	Colon cancer Caco-2 cells		IP6	Proliferation inhibition after 24 h incubation in 1–10 mM IP6 (*p* < 0.05); after 48 h incubation proliferation inhibition was statistically significant in 5–10 mM IP6 Higher apoptotic index in IP6-treated cells compared to the controls Higher expression of p21^Waf1/Cip1^ and higher Caspase-3 activity in IP6-treated cells Reduction of AKT-1 activity and p70S6K in IP6-treated cells
Schröterová et al. (2018) [55]	Colon cancer SW620 cells		IP6	Significant decrease in cell migration at all IP6 concentrations (dose-dependent) Significant decrease in the levels of MMP-2 and MMP-9 after 12, 24, and 48 h exposure to all IP6 concentrations Decreased level of I-Cam1 after 12 and 24 h exposure to 1 mM IP6 and decreased level of EpCam after 48 h exposure to 1 mM IP6, decreased level of N-cadherin after 12 and 48 h exposure to all IP6 concentrations

**Table 4:** Antineoplastic activity of cyclitols in other cancers.

Type of Cancer	Study	Clinical Status	Cyclitol	Results
Prostate	Shamsuddin et al. (1995) [62]	PC-3 human prostate cells	IP6	Significant growth inhibition in 1 mM IP6 concentration after 24 h, and in 0.1 mM IP6 after 72 h Significant decrease in DNA synthesis after 3 h in 1 mM IP6 concentration Increased expression of HLA-1 class antigen in 1 mM IP6 and in 5 mM IP6 Significant increase in prostatic acid activity
Prostate	Sharma et al. (2003) [64]	Mouse prostate (TRAMP-C1) cells	IP6	The cell treatment with 1–4 mM of IP6 resulted in significant cell growth inhibition, increased cell death, and increased apoptosis IP6, at a concentration of 1–4 mM, induced G0–G1 phase arrest of the treated cells
Prostate	Singh et al. (2004) [65]	DU145 cells injected into nude mice	IP6	Significant reduction in tumor weight in mice treated with 2% of IP6 Decreased proliferation index and increased apoptotic index in IP6-treated cells (1–2% IP6) Inhibition of microvessel density in prostate tumor xenografts
Prostate	Lin et al. (2013) [63]	PC-3 and DU145 cells	d-pinitol	Significant decrease in cell migration and invasion after treatment with 3–30 mM d-pinitol Significant decrease in integrin αvβ3 expression after treatment with 3–30 mM d-pinitol Significant decrease in the levels of p-FAK a p-p65 and in the activity of c-Src kinase and NF-κβ luciferase after d-pinitol treatment
Pancreas	Somasundar et al. (2004) [66]	MIAPACA and PANC1 pancreatic cancer cell lines	IP6	Significant growth inhibition of cancer cells in all concentrations of IP6 Increased apoptotic rate in MIAPACA in 2.5 mM of IP6 and in PANC1 in 5 mM of IP6
Pancreas	McMillan et al. (2007) [67]	PANC1 and MIAPACA	IP6	Significant reduction of proliferation in the IP6-treated group (synergistic effect with catechin, after 48 and 72 h incubation) Synergistic effect with catechin in increasing apoptosis (alone IP6 did not induce a statistically significant reduction) Significant reduction of the VEGF level in IP6-treated cells (synergistic effect with catechin)
Liver	Lee et al. (2005) [68]	Rat treated with DEN	IP6 and Ins	Significant reduction in preneoplastic lesions in every experimental group (synergistic effect noted between IP6 and Ins) Significant increase in glutathione-S-transferase activity and a significant decrease in catalase activity in every experimental group (a synergistic effect between IP6 and Ins was noted) Significantly lower level of lipid peroxidation in experimental groups
Liver	Nishino (2009) [69]	Patients with chronic viral hepatitis and cirrhosis	MI	81% inhibition of liver cancer in the experimental group compared to controls
Osteosarcoma	Ren et al. (2017) [70]	In vitro (K7M2 and MG63.3 cells) and in vivo trials (mice with injected K7M2 cells)	IP6	Treatment of K7M2 cells with 0.3 mM IP6 and MG63.3 cells with 4 mM of IP6 resulted in 50% proliferation inhibition Inhibition of cancer cell growth In each line, exposure to 5 mM of IP6 for 6 h resulted in a significantly increased level of caspase 3/7 In the PuMA model, IP6 treatment of both K7M2/GFP and MG63.3/GFP resulted in markedly inhibited metastatic outgrowth in lung tissue IP6 treatment (60 mg/kg) of mice injected with K7M2 cells resulted in significantly higher survival rates (120 days versus 72 days) but did not influence the osteosarcoma primary tumor growth rate
Melanoma	Khurana et al. (2018) [71]	Case report	IP6 + Ins	Complete clinical and radiological remission after three years of treatment (primary patients diagnosed with stage IIIB melanoma had two reoccurrences of melanoma)

## Abbreviations

IL-6	interleukin-6
STAT	signal transducer and activator of transcription gene family
BALF	bronchoalveolar lavage fluid
Akt	RAC-alpha serine/threonine–protein kinase
ERK	extracellular signal-regulated kinase
FEC-5	fluorouracil, epidoxorubicin and cyclophosphamide
CMF	cyclophosphamide methotrexate fluorouracil
HLA-1	human leukocyte antigen I
P-FAK	phosphorylated focal adhesion kinase
BMI body	mass index
HOMA-IR	homeostatic model assessment for insulin Resistance
